# Sanguinarine suppresses migration and metastasis in colorectal carcinoma associated with the inversion of EMT through the Wnt/β‐catenin signaling

**DOI:** 10.1002/ctm2.1

**Published:** 2020-04-14

**Authors:** Man Zhu, Zhengyan Gong, Qing Wu, Xianpeng Shi, Qi Su, Yanmin Zhang

**Affiliations:** ^1^ School of Pharmacy Health Science Center Xi'an Jiaotong University Xi'an P.R. China

**Keywords:** colorectal cancer, EMT, metastasis, sanguinarine, Wnt/β‐catenin

## Abstract

**Background:**

Unresectable lung or liver organ metastases of colorectal carcinoma (CRC) remain a major obstacle in clinical therapeutics. Epithelial to mesenchymal transition (EMT), a major cause of highly frequent metastasis in tumor, can be promoted by the Wnt/β‐catenin pathway that is aberrantly activated in approximately 90% of CRC. This research aimed to elucidate the antimetastatic potential of sanguinarine (SG) in CRC and the underlying molecular mechanism.

**Methods:**

The in vitro anticancer effect of SG was determined via cell viability experiment and colony formation assay. Xenograft model of nude mice was used to confirm the antitumor effect of SG in vivo. The antimetastatic potential of SG was investigated by the metastasis model of nude mice, hematoxylin and eosin (H&E) staining, migration assay, and wound‐healing analysis. Immunoblotting analysis, immunofluorescence staining, and immunohistochemistry assay were conducted to elucidate the molecular mechanism.

**Results:**

In this study, we reported that SG has a selective inhibitory effect on LoVo cells with metastatic characteristics. Furthermore, our results showed attenuation in the migration and metastatic ability of SG‐treated LoVo cells and also decreased metastatic nodules of liver and lung in mice metastasis model. This was also confirmed at the molecular level via H&E staining. Further study revealed that SG had negative impacts on the Wnt/β‐catenin pathway and EMT markers in LoVo cells both in vitro and in vivo.

**Conclusions:**

Taken together, the antimetastatic potential of SG attributed to the suppression of the Wnt/β‐catenin signaling, which further prevented EMT progression. SG may be of value in a potential therapy for the management of metastasis CRC.

**Abbreviations**
APC: adenomatous polyposis coli protein
CK1α: casein kinase 1‐alpha
CRC: colorectal carcinoma
DMEM: Dulbecco's modified Eagle's medium
DMSO: dimethyl sulfoxide
ECL: enhanced chemiluminescent
EMT: epithelial to mesenchymal transition
GSK‐3β: glycogen synthase kinase‐3 beta
H&E: Hematoxylin and eosin
SG: sanguinarine

## 1 BACKGROUND

Colorectal carcinoma (CRC) is one of the most prevalent malignancies with high http://morbidity and mortality worldwide. Distant metastasis is the main obstacle in cancer treatment and is one of the leading causes of death among CRC patients.^1^ In CRC, metastatic progression often occurs in hepar, lung, and lymph nodes.^2^ Although recent treatment approaches including chemotherapy, surgery, and radiotherapy have been greatly improved, patients with metastases are always insensitive to the existing therapies, which leads to high mortality in CRC.^3^ There is a constant demand for a new treatment for CRC therapies. Hence, a better approach of targeting proliferation and metastasis for CRC treatment is required.

The Wnt family is constituted by a group of cysteine‐rich glycoproteins that can initiate varied signaling cascades such as the planar cell polarity pathway, the canonical pathway, and the Wnt/Ca^2+^ pathway.^4^ Typically, initiation and progression of CRC are closely associated with deregulation of the canonical Wnt/β‐catenin pathway. It has been reported that over 90% CRC patients are diagnosed with mutation and/or overexpression of Wnt pathway components (ie, adenomatous polyposis coli protein [APC] and β‐catenin).^5^ At a molecular level, the major expression of β‐catenin in cytoplasm or nucleus depends on a cytoplasmic destruction complex including AXIN1/2, glycogen synthase kinase‐3 beta (GSK‐3β), APC, and casein kinase 1‐alpha (CK1α). Aberrant Wnt pathway components disrupt such complex and lead to plenty of β‐catenin accumulating in the cytoplasm, translocating to the nucleus, and contributing to carcinogenesis.^6^

There is a general acceptance that Wnt/β‐catenin pathway could give rise to the process of epithelial to mesenchymal transition (EMT), finally triggering CRC metastasis.^7^ EMT describes a process whereby polarized epithelial cells detach from tight junctions and transform to mesenchymal cells, which is accompanied by prominent changes of cell shape, polarity, and migration. The acquisition of mesenchymal cell trait is not only a prerequisite but also the initial event in cascade, which drives a key mechanism in the invasiveness, migration, and metastasis in vast cases of carcinoma.^8^ EMT‐associated transcription factors Snail, Twist, and Zeb take a vital part in EMT progression through repressing epithelial genes such as http://E-cadherin and activating mesenchymal genes such as http://N-cadherin.^9^ Studies have determined that translocation of β‐catenin is conducive to the occurrence of EMT by means of inducing transcription of Snail and Twist and inhibiting E‐cadherin expression.^10^, ^11^ These findings indicate that the excessive excitation of the Wnt/β‐catenin pathway may behave as an initiating event and a primary transforming event of colorectal tumorigenesis. There is no doubt that blocking this pathway is a critical approach for CRC treatment.

**FIGURE 1:**
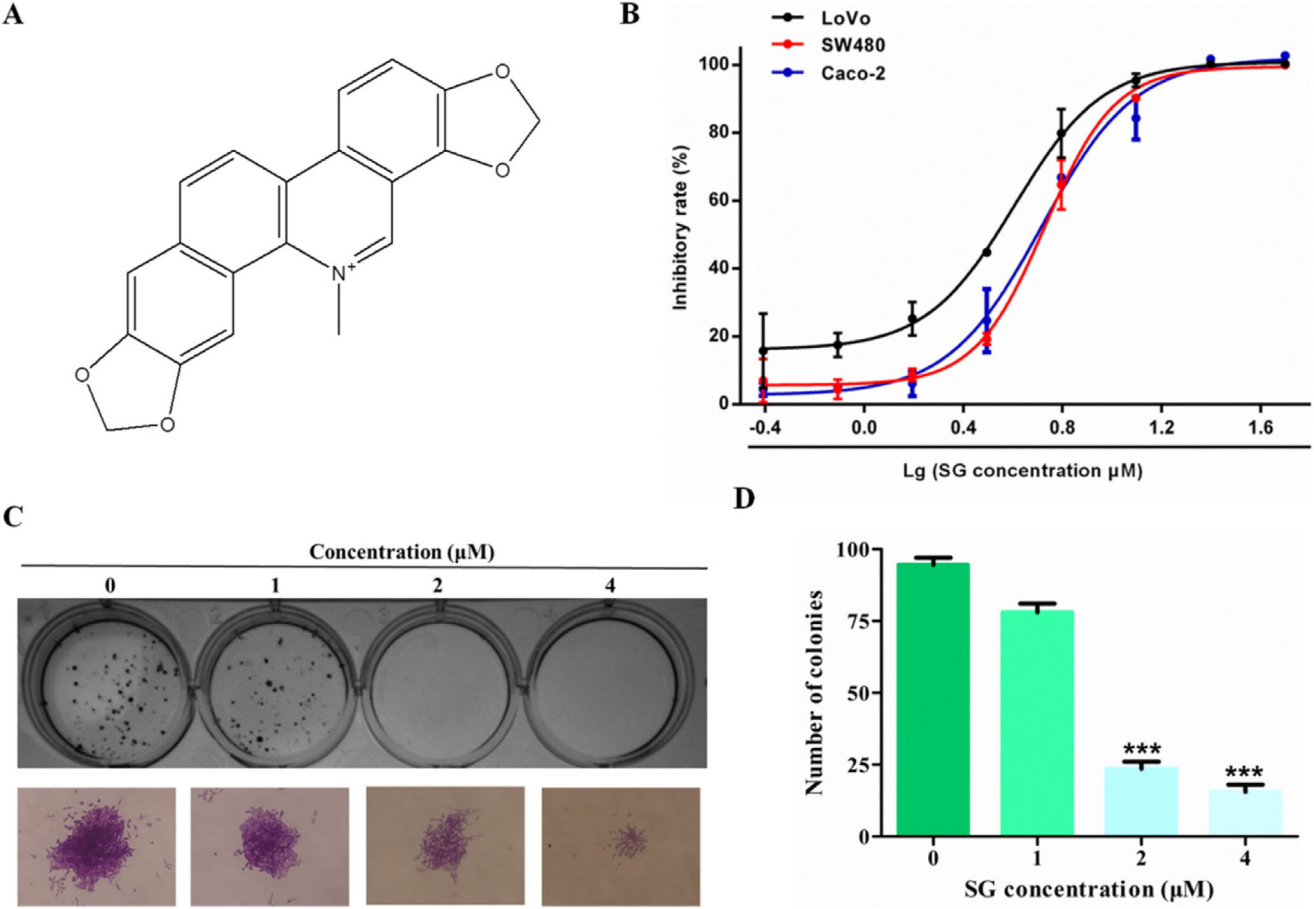
Sanguinarine (SG) shows a prominent inhibitory effect on LoVo cells in vitro. A, The chemical structure of SG. B, Effects of SG on cell proliferation in LoVo, SW480, and Caco‐2 cells were determined by MTT assay. Cells were treated with SG (0.39‐50 µM) for 48 hours. C, Effects of SG on colony formation in LoVo cells (200×). D, Quantification of the data represented in (C). Values are presented as mean ± SD. *P < .05, **P < .01, and ***P < .001 versus the control group

Naturally occurring plant‐based agents are a rich source of valuable antineoplastic resources. Natural alkaloid sanguinarine (SG) (Figure 1A) is a bioactive benzophenanthridine alkaloid mainly extracted from the plants of Papaveraceae family, such as blood root plants *Chelidonium majus*, *Sanguinaria canadensis*, and *Argemone mexicana*. It exerts broad‐spectrum pharmacological activities including antimicrobial, anti‐angiogenic and anti‐inflammatory properties.^12^ Notably, other documented properties of SG are its growth inhibitory activities on a range of cancer types. For example, it causes cell cycle arrest and apoptosis in hepatocellular carcinoma based on the restoration of miR‐16 tumor suppressor function.^13^ However, there are no reports mentioned for the antimetastasis activity of SG. In this study, we aimed to elucidate the antimetastatic potential of SG in CRC and the underlying molecular mechanism which is by modulating the Wnt/β‐catenin signaling. We suggest that SG could be a novel therapeutic agent for CRC.

## 2 MATERIALS AND METHODS

### 2.1 Chemical and reagents

SG (HPLC ≥ 99%) was purchased from Baoji Herbest Biotech Co. Ltd (Shaanxi, China). Dulbecco's modified Eagle's medium (DMEM), PBS, fetal bovine serum (FBS), antibiotics, and trypsin were purchased from HyClone (Logan, UT). 3‐(4,5‐Dimethylthiazol‐2‐yl)‐2,5‐diphenyl‐2H‐tetrazolium bromide (MTT) powder was obtained from Sigma‐Aldrich (St. Louis, MO). Crystal violet was purchased from Beijing Chemical Works (Beijing, China). Dimethyl sulfoxide (DMSO) and paraformaldehyde were obtained from Tianjin Dibo chemical Co. Ltd (Tianjin, China). RIPA Lysis Buffer, BCA protein assay reagent kit, and 4′,6‐diamidino‐2‐phenylindole were obtained from Pioneer Biotechnology Co. Ltd (Shaanxi, China). Enhanced chemiluminescent (ECL) plus reagent kit was purchased from 4A Biotech Co. Ltd. (Beijing, China). Protease and phosphatase inhibitors were obtained from Roche Tech. (Basel, Switzerland). MMP2 and MMP9 rabbit mAb were from ABclonal (Boston, MA). β‐Catenin and p‐β‐catenin rabbit mAb were obtained from Cell Signaling (Boston, MA). AXIN1, GSK‐3β, p‐GSK‐3β, E‐cadherin, APC, N‐cadherin, and Snail mAb were purchased from Protein Technology Group (Chicago, IL).

### 2.2 Cell line and cell culture

LoVo, SW480, and Caco‐2 human CRC cell lines were all obtained from the Shanghai Institute of Cell Biology at the Chinese Academy of Sciences. All the cell lines were maintained in DMEM medium supplemented with penicillin and streptomycin and grown in an incubator with 5% CO_2_ at 37°C. DMEM medium used in LoVo and SW480 cell culture is supplemented with 10% FBS, whereas the medium is supplemented with 20% FBS in Caco‐2 cell culture.

### 2.3 Cell viability assay

MTT method was used to test cell viability. Growing cells were cultured in 96‐well plates with 180 µL cell suspension per well for 24 hours. Followed by an increased gradient of SG (0.39, 0.78, 1.56, 3.12, 6.25, 12.50, 25.00, and 50.00 µM) treatment, the cells were maintained in the incubator. After 48 hours, the medium with SG was substituted by 180 µL serum‐free medium with 20 µL MTT solution per well. After 4 hours of incubation, the culture supernatant was discarded and DMSO (150 µL) was added followed by shaking for 15 minutes. The plate was measured by a Bio‐Rad microplate reader (Hercules, CA).

### 2.4 Colony formation assay

LoVo cells were cultured in 12‐well plates. After 24 hours, increased gradient of SG (0, 1, 2, 4 µM) was added and were maintained for 48 hours. Next, the medium containing SG was replaced by a fresh complete DMEM medium. After growth in the plates for 14 days, the colonies were visible and rinsed twice with PBS and fixed with 70% methanol. After 15‐minute incubation, the colonies were rinsed twice with PBS buffer and stained by crystal violet (0.2%). Images of each well were taken by an inverted fluorescence microscope.

### 2.5 Animal studies

Female nude mice (4‐6 weeks of age) were used to conduct all the in vivo studies. The mice were housed at Laboratory Animal Center of Xi'an Jiaotong University in a specific pathogen‐free atmosphere. All the mice studies were performed according to regional authority guidelines.

For the xenograft model, the mouse was subcutaneously inoculated with 200 µL LoVo cell suspension (2 × 10^7^ cells/mL) at right flanks. The tumor volume was measured once every two days as (*A* × *B* ^2^)/2, where *A* is the longer diameter and *B* is the shorter diameter. The mice were randomly grouped into four groups (four mice in each group) and administered with SG (1.25, 2.5, or 5 mg/kg) or 0.5% CMC‐Na solution orally. Body weight and tumor volume of each mouse were registered daily. Following 14 days of continuous treatment, the mice were euthanized. The tumors and spleens were collected and weighed.

For the metastasis model, each mouse was injected to the tail veins with 200 µL LoVo cell suspension (1 × 10^7^ cells/mL). After 7 days, mice were randomly divided into two groups (three mice in each group) and administered with 2.5 mg/kg SG or 0.5% CMC‐Na solution orally. Body weight of each mouse was monitored daily. After 28 days of continuous treatment, the mice were sacrificed. The liver and lung were collected and weighed for in vivo tumor invasion and metastasis analysis. Metastatic liver/lung nodules were counted and further confirmed via hematoxylin and eosin (H&E) staining.

### 2.6 Migration assay

Cells suspended in complete medium were cultured in the upper chamber, and the lower chamber was full of 500 µL complete medium. After 24‐hour incubation, an increased gradient of SG (0, 1, 2, 4 µM) was added into the upper chamber and conserved for 48 hours. For SG and XAV939 combination experiment, the cells were exposed to 2 µM SG and/or 5 µM XAV939 for 48 hours. Next, the medium with drugs in the upper chamber was substituted with serum‐free medium, and the medium in the lower chamber was substituted with a complete medium containing 20% FBS. Following incubation at indicated hours, noninvading cells on the upper membranes were discarded carefully. The migrated or invaded cells on the lower membranes were fixed with 95% ethanol for 15 minutes and stained with 0.1% crystal violet.

### 2.7 Wound‐healing assay

Cells were cultured in 12‐well plates overnight. A sterile P200 pipet tip was used to create a straight scratch in the cell monolayer. After rinse with PBS buffer twice, the plate was incubated with fresh complete DMEM medium containing increased gradient of SG (0, 1, 2, 4 µM) for 24 hours. For SG and XAV939 combination experiment, the cells were exposed to 2 µM SG and/or 5 µM XAV939 for 24 hours. The plate was imaged at 0 hour and 24 hours by an inverted fluorescence microscope.

### 2.8 Immunoblotting analysis

For SG treatment experiment, the cells were exposed to increased gradient of SG (0, 1, 2, 4 µM). For SG and XAV939 combination experiment, the cells were exposed to 2 µM SG and/or 5 µM XAV939. Following 48‐hour incubation, the treated cells were rinsed using PBS buffer and incubated in RIPA buffer on ice for 30 minutes. The product was collected and the ultrasonic crushing method was used to break the cells. Then the product was centrifuged at 4°C for 10 minutes. After the measurement of the protein concentrations via a BCA Protein Quantification kit, 5× loading buffer with bromophenol blue was added to the protein supernatant. Following separation on 12% SDS‐polyacrylamide gel, the proteins were then transferred from the gel to PVDF membranes. Following incubation with 10% non‐fat milk for 2 hours, the membranes were treated with primary antibodies for 12 hours at 4°C. After incubation with HRP‐conjugated secondary antibodies for 1 hour at 37°C, the membranes were then rinsed four times in tris buffered saline tween. Finally, the reactive bands were tested by a Tanon 5200 automatic chemiluminescence image analysis system using an ECL kit.

### 2.9 Immunofluorescence staining

The SG‐treated (2 µM) LoVo cells were incubated in 4% paraformaldehyde solution. After three washes in PBS, the cells were blocked by 10% BSA solution at room temperature. Then β‐catenin first antibody was added and maintained for 4 hours at 37°C. Following incubation with fluorescein isothiocyanate‐conjugated secondary anti‐rabbit IgG antibody, the cells were labeled by DAPI and images were captured.

### 2.10 Immunohistochemistry assay

Tumor tissues were incubated in 4% paraformaldehyde and embedded in paraffin. The paraffin samples were sectioned as thick as 5 µm, and then were deparaffinized by xylene. After dehydration in a decreased gradient of ethanol, the samples were then incubated with citrate buffer and immersed in 3% H_2_O_2_ solution for 10 minutes. Thereafter, the corresponding primary antibodies were applied on the samples at 4°C overnight. Following incubation with the HRP‐conjugated secondary antibodies, the sections were incubated with diaminobenzidine and re‐dyed with hematoxylin for capturing images.

### 2.11 H&E staining

The liver or lung tissues embedded in paraffin were deparaffinized as mentioned above and stained with hematoxylin for 10 minutes. After treatment with hydrochloric acid alcohol solution and ammonium hydroxide each for 30 seconds, the samples were then stained with eosin for 3 minutes. The increased concentration of alcohol was used to dehydrate the sections. Then the sections were treated with xylene three times each for 3 minutes. Finally, neutral balsam was used for section mounting.

### 2.12 Statistical analysis

The values were evaluated as mean ± standard error of means of three independent experiments. Statistical analysis was conducted utilizing SPSS (version 18.0). Student's *t*‐test was used to compare individual data with the control values. Analysis of variance was utilized to analyze statistical differences between groups under different conditions. Significance values were set at *P < .05, **P < .01, ***P < .001, ^#^ *P *< .05, ^##^ *P *< .01, and ^###^ *P *< .001.

## 3 RESULTS

### 3.1 SG shows a prominent inhibitory effect on colon cancer cells in vitro

To evaluate the anticancer effect of SG on CRC, three CRC cell lines, SW480, LoVo, and Caco‐2, were exposed to increased concentration of SG (0.39‐50.00 µM) for 48 hours. As shown in Figure 1B, SG showed a good inhibitory effect in all the tested CRC cells. In line with this result, LoVo cells were most sensitive to SG with an IC_50_ value of 4.03 µM, whereas the IC_50_ values of SW480 and Caco‐2 cells were 5.37 and 5.20 µM, respectively. Thus, LoVo cells were selected for the following study on SG treatment mechanism. Similar results were obtained from the colony formation analysis; the colony size and number of LoVo cells were strikingly decreased in a dose‐dependent manner in comparison to the control group (Figure 1C,D).

### 3.2 In vivo inhibitory effect of SG on LoVo cells

**FIGURE 2:**
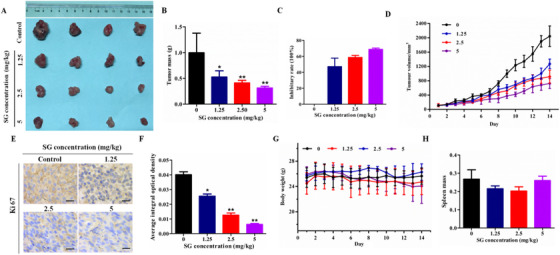
In vivo inhibitory effect of SG on LoVo cells. A, Photographs of control and SG‐treated group tumors. B, Effect of SG on the tumor mass. C, Effect of SG on tumor inhibitory rate. D, Tumor volume change throughout the study. E, Ki67 immunochemistry (200×). F, Histogram represents the statistical analysis of (E). G, Body weight change throughout the study. H, Effect of SG on spleen mass. Values are presented as mean ± SD. *P < .05, **P < .01, and ***P < .001 versus the control group

To extend the in vitro study to in vivo tumor growth, LoVo cells xenograft model of nude mice was constructed and the anti‐tumor effect of SG was evaluated. The results were similar to the in vitro experiments that SG could suppress LoVo tumor growth both on tumor mass and volume (Figure 2A,B, and D). Such inhibitory effect was enhanced in a dose‐dependent manner so that the average inhibitory rate reached 47.12%, 58.65%, and 68.67% in xenograft tumors following SG gavage as 1.25, 2.5, and 5 mg/kg (Figure 2C). Moreover, the reduction of Ki67, a proliferation marker, in tumor tissues following SG treatment also confirmed the above results at a molecular level (Figure 2E,F). Notably, there was not a prominent body weight and spleen index reduction in all the in vivo experiments (Figure 2G,H).

### 3.3 SG blocks the migration ability of LoVo cells

**FIGURE 3:**
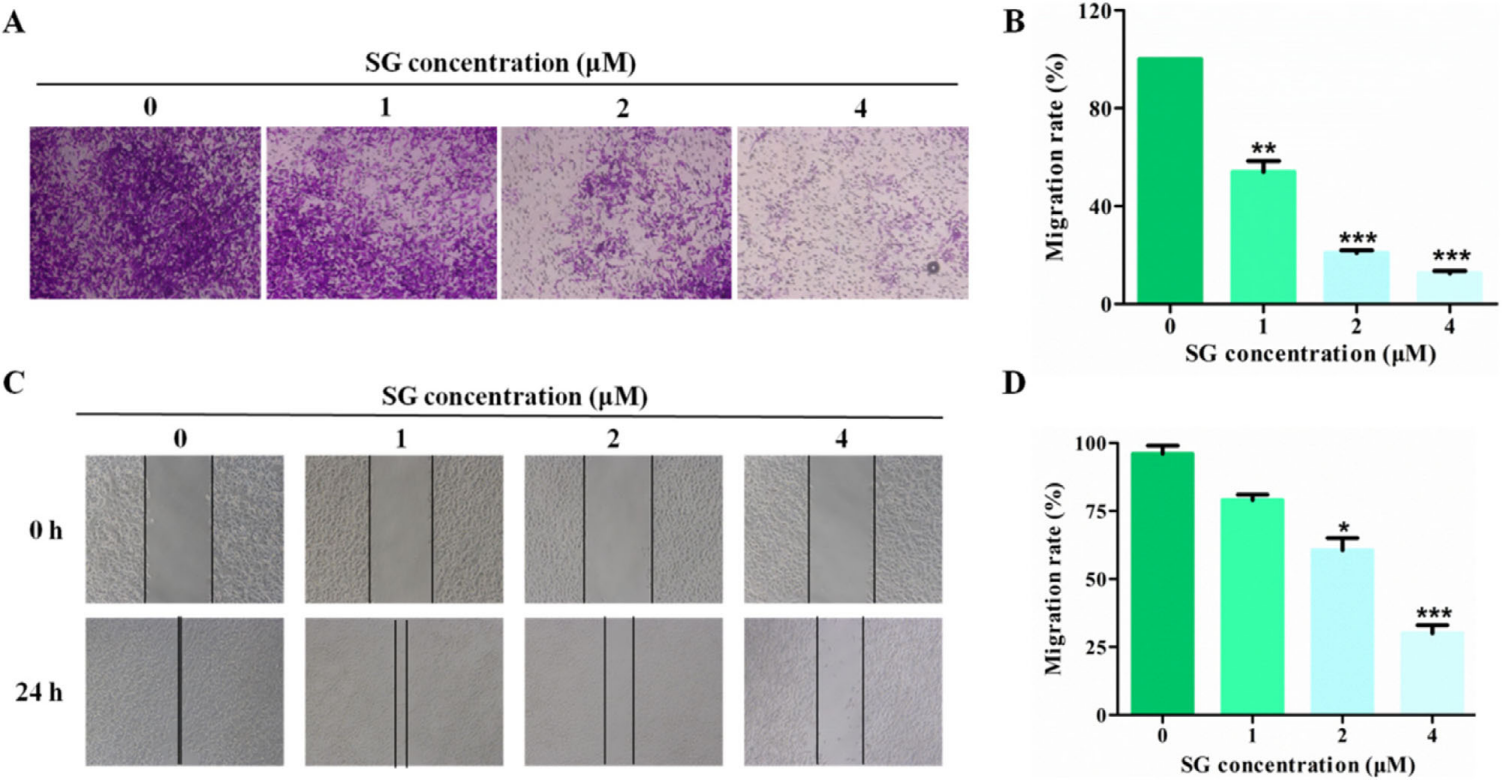
SG blocks the migration ability of LoVo cells. A, Transwell assays were conducted to observe the migratory cells in control and SG‐treated LoVo cells (200×). B, Histogram represents the statistical analysis of (A). C, The migration rate of control and SG‐treated LoVo cells observed through wound‐healing assays (200×). D, Histogram represents the statistical analysis of (C). Values are presented as mean ± SD. *P < .05, **P < .01, and ***P < .001 versus the control group

Since LoVo cells are characterized by high invasiveness, we thus investigated the influence of SG on LoVo cell migration ability. The number of migration cells in SG treatment groups was reduced significantly compared with the control group (Figure 3A,B). To further elucidate the inhibitory effect of cell migration of SG, we also conducted wound‐healing experiment. Similar results on migration inhibition were noted in wound‐healing assays. LoVo cells exposed to SG displayed delayed closure of wound gaps as observed at 24‐hour intervals compared with control cells (Figure 3C,D).

### 3.4 SG suppresses the metastasis of LoVo cells in vivo

**FIGURE 4:**
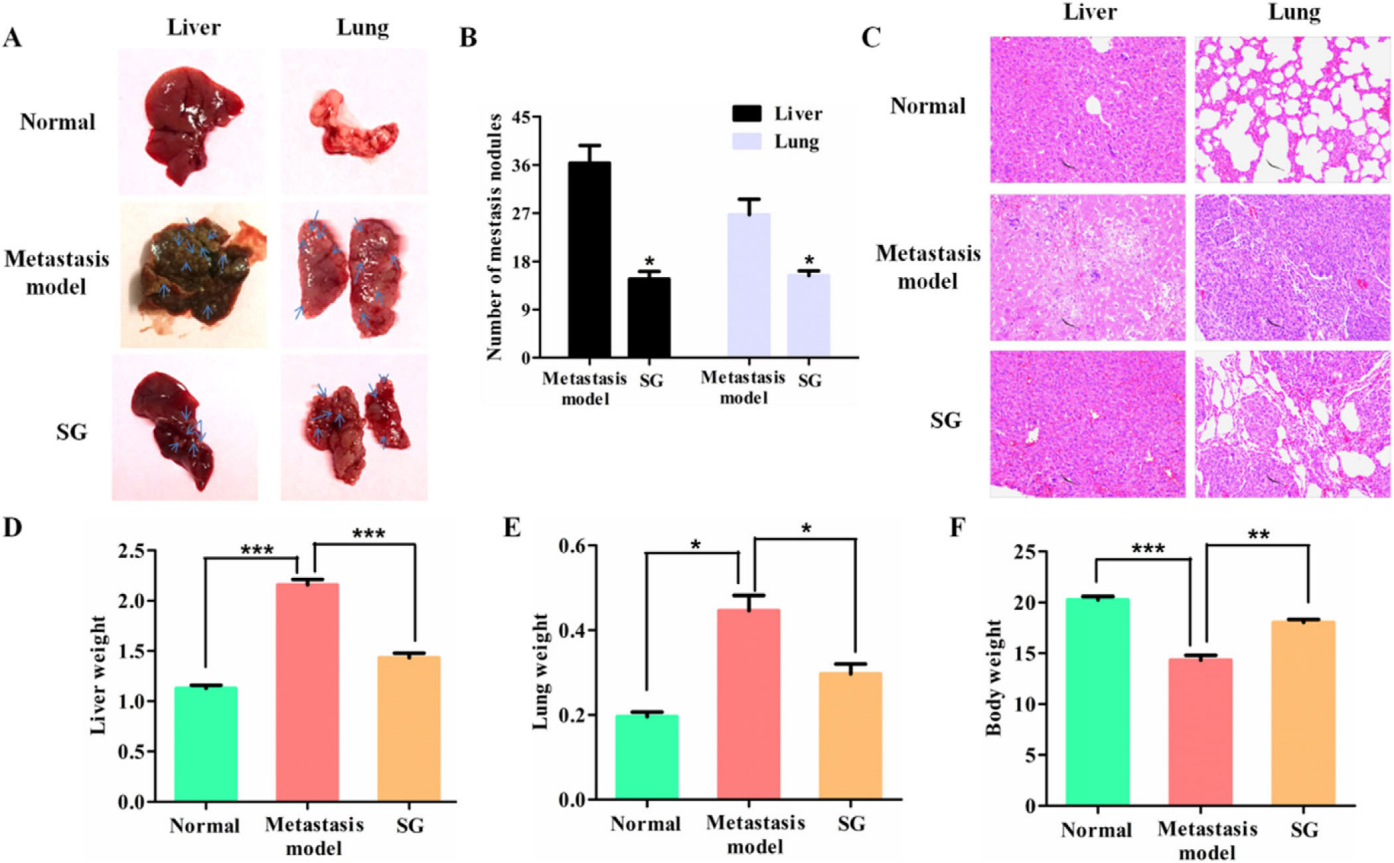
SG suppresses the metastasis of LoVo cells in vivo. A, Presence of hepatic and pulmonary metastatic nodules in nude mice 5 weeks after tail vein injection of the tumor cells. The swollen and obvious metastatic lesions are indicated by the blue arrow. B, The number of metastatic nodules in the liver and lung. C, Representative images of the histological assessment of the lungs and livers via H&E staining (200×). D, Average weights of livers in each group. E, Average weights of lungs in each group. F, Average body weights in each group. Values are presented as mean ± SD. *P < .05, **P < .01, and ***P < .001 versus the indicated group

**FIGURE 5:**
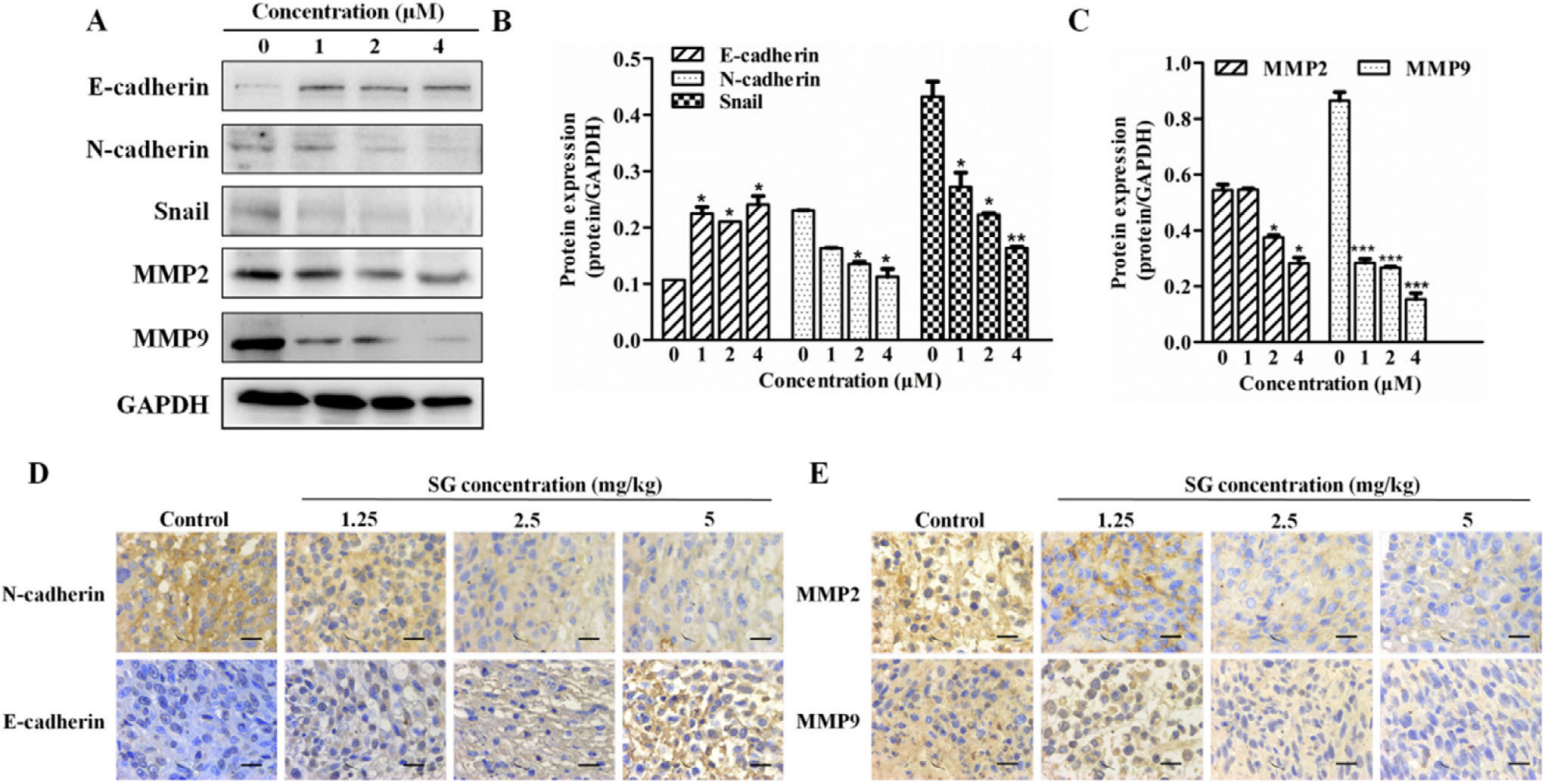
SG regulates EMT‐related molecules. A, Protein expression of E‐cadherin, N‐cadherin, Snail, MMP2, and MMP9 in LoVo cells treated with SG for 48 hours. B, Quantification of the protein expression of E‐cadherin, N‐cadherin, and Snail represented in (A). C, Quantification of protein expression of MMP2 and MMP9 represented in (A). D, Immunochemistry assay of E‐cadherin and N‐cadherin in tumor tissues (200×). E, Immunochemistry assay of MMP2 and MMP9 in tumor tissues (200×). Values are presented as mean ± SD. *P < .05, **P < .01, and ***P < .001 versus the control group

To validate our findings in vitro, we established the metastasis mice model by means of tail vein injection of LoVo cells. After mice euthanization at 5 weeks postinoculation, lung and liver organs were obtained as shown in Figure 5A. Both livers and lungs of metastasis models were filled with metastatic nodules compared with the normal mice, whereas the group that received SG treatment exhibited fewer metastatic lesions (Figure 4A,B). H&E staining of livers and lungs with LoVo cells also showed greater tumor metastasis compared with SG treatment groups (Figure 4C). Furthermore, the weights of the liver and lung in the metastasis model were much higher than that of the SG treatment group, while metastasis models lost a lot of body weight as compared to the SG treatment group (Figure 4D‐F).

### 3.5 SG regulates EMT‐related molecules

EMT is essential for migration and distant organ metastasis of tumor cells. The effect of SG on EMT‐related molecules was evaluated by western blotting and immunohistochemistry assay. The expression of E‐cadherin, a key molecule for EMT, was significantly increased after treatment by SG at indicated doses (Figure 5A,B). N‐cadherin is another important cadherin and its increased expression represents the occurrence of EMT. The protein level of N‐cadherin and the transcription factor Snail produced during EMT process were both suppressed by SG in LoVo cells (Figure 5A,B). The result of the immunohistochemistry assay in Figure 5D indicated that SG could upregulate the expression of E‐cadherin and decrease the protein level of N‐cadherin in the colorectal tumor tissues.

Metalloproteinases (MMPs) could degrade the extracellular matrix and promote the migration of tumor cells. MMP2 and MMP9 were essential members of MMPs family, which could be secreted during the EMT process, and the protein level of the two members was also examined by immunoblotting (Figure 5A,C). Both the molecules in the SG‐treated LoVo cells were significantly downregulated in comparison to that in the control group. Moreover, gavage administration of SG inhibited the protein level of MMP2 and MMP9 in the tumor tissues of the xenograft model (Figure 5E).

### 3.6 SG regulates Wnt/β‐catenin signaling

**FIGURE 6:**
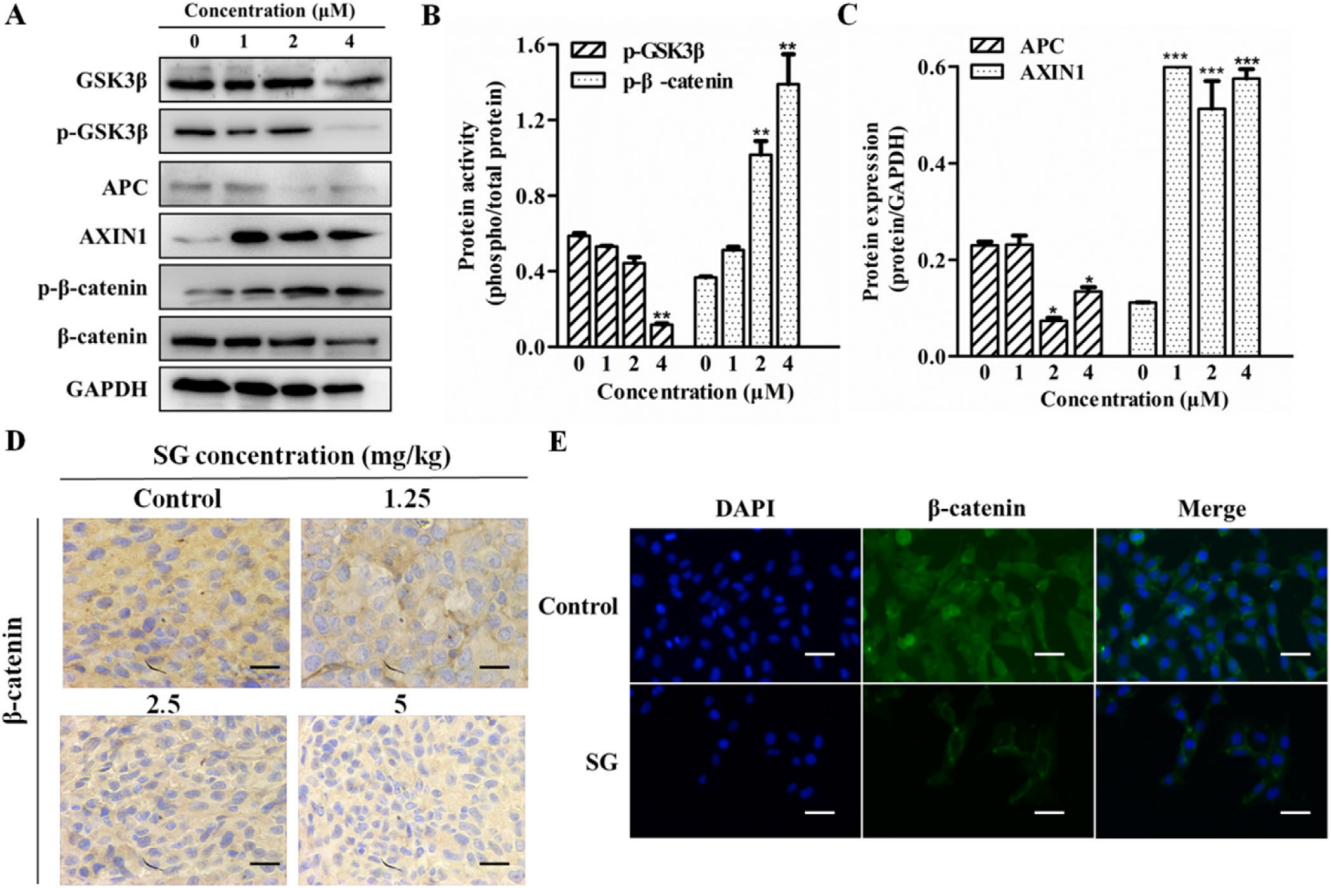
SG regulates Wnt/β‐catenin signaling pathway. A, Protein expression of GSK‐3β, p‐GSK‐3β, APC, AXIN1, p‐β‐catenin, and β‐catenin in LoVo cells treated with SG for 48 hours. B, Quantification of the protein expression of p‐GSK‐3β and p‐β‐catenin represented in (A). C, Quantification of the protein expression of APC and AXIN1 represented in (A). D, Immunochemistry assay of β‐catenin in tumor tissues (200×). E, Immunofluorescence analysis of the β‐catenin protein in LoVo cells treated with SG (200×). β‐Catenin (green), DAPI (blue) staining, and merged images indicate the nuclear translocation and expression of β‐catenin. Values are presented as mean ± SD. *P < .05, **P < .01, and ***P < .001 versus the control group

The aberrant activation of the Wnt/β‐catenin signaling is frequent in CRC, which could promote EMT and metastasis of tumor cells. EMT‐related molecules E‐cadherin, Snail, and MMPs were downstream signaling of the Wnt/β‐catenin signaling. Based on our previous study, we proposed a hypothesis that the Wnt/β‐catenin pathway could be suppressed by SG. The results in Figure 6A‐C confirmed our conjecture. SG remarkably increased the protein level of AXIN1 and the phosphorylation of β‐catenin, resulting in the decreased total expression of β‐catenin. Moreover, the protein level of APC and p‐GSK‐3β were significantly suppressed by SG. Immunofluorescence staining of β‐catenin was conducted and the result is shown in Figure 6E. Green fluorescence represented β‐catenin and distributed throughout the cell at a high intensity in the control group. However, the fluorescent intensity was decreased in SG‐treated LoVo cells and was much lower in the nucleus than that in the cytoplasm, indicating that SG could not only decrease β‐catenin expression but also suppress its translocation in the nucleus. This result was further confirmed by the immunohistochemistry assay in the xenograft models, as shown in Figure 6D.

### 3.7 SG reverses EMT progression through Wnt/β‐catenin signaling

**FIGURE 7:**
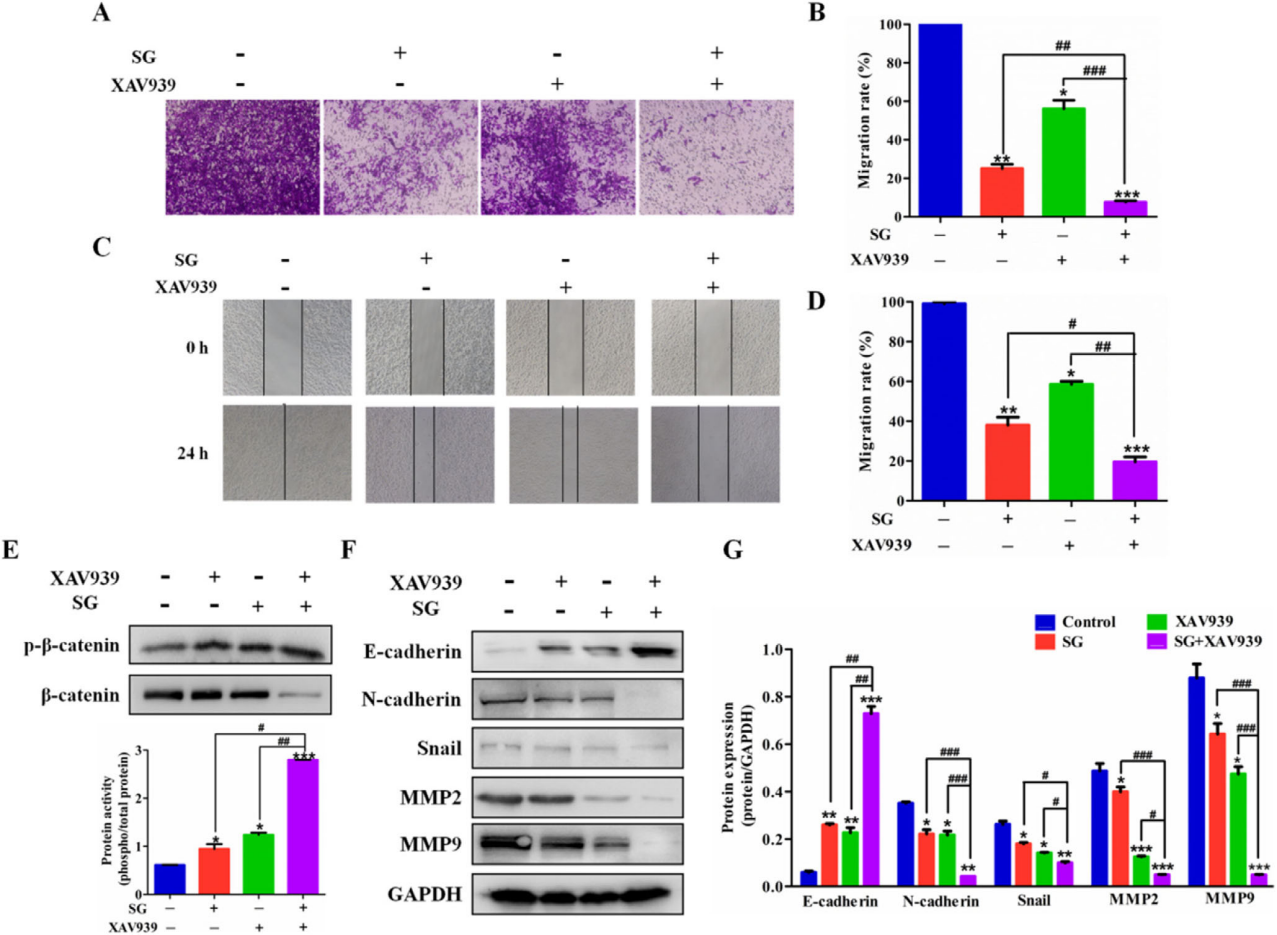
SG reverses EMT progression through Wnt/β‐catenin signaling pathway. A, Transwell assays were conducted to observe the migratory cells in control, SG‐treated, XAV939‐treated, or combination treatment LoVo cells (200×). B, Histogram represents the statistical analysis of (A). C, The migration rate of control, SG‐treated, XAV939‐treated, or combination treatment LoVo cells observed through wound‐healing assays (200×). D, Histogram represents the statistical analysis of (C). E, Top: Protein expression of p‐β‐catenin and β‐catenin in LoVo cells treated with SG, XAV939, or combination for 48 hours. Bottom: Quantification of the protein expression of p‐β‐catenin. F, Protein expression of E‐cadherin, N‐cadherin, Snail, MMP2, and MMP9 in LoVo cells treated with SG, XAV939, or combination for 48 hours. G, Quantification of the protein expression of E‐cadherin, N‐cadherin, Snail, MMP2, and MMP9 represented in (F). Values are presented as mean ± SD. *P < .05, **P < .01, and ***P < .001 versus the control group; *^#^P <* .05, *^##^P* < .01, and *^###^P* < .001 versus the indicated group

Next, an effective inhibitor of the Wnt/β‐catenin signaling XAV939 was utilized to confirm that SG‐induced inversion of EMT may involve Wnt/β‐catenin signaling. Our results indicated that an enhanced inhibitory effect was observed in cell migration ability in LoVo cells exposed to SG and XAV939 compared to their individual exposure (Figure 7A,B). Furthermore, the migration of concurrent treatment LoVo cells with SG and XAV939 into the wound was much slower than that of the cells exposed to a single agent alone (Figure 7C,D).

A combination of XAV939 and SG was further used to elucidate whether SG was through suppression of the Wnt/β‐catenin signaling to regulate the EMT and MMPs signaling. LoVo cells exposed to XAV939 demonstrated higher phosphorylation of β‐catenin compared to the cells in the control group, demonstrating the suppression of the Wnt/β‐catenin signaling (Figure 7E). The EMT signaling and MMPs were also regulated by XAV939, with obvious increased E‐cadherin and decreased N‐cadherin, Snail, MMP2, and MMP9 (Figure 7F,G). Moreover, co‐administration of XAV939 and SG exerted synergistic effect in the suppression of β‐catenin and regulation of the downstream migration‐related molecules such as E‐cadherin, N‐cadherin, Snail, MMP2, and MMP9 (Figure 7E‐G).

## 4 DISCUSSION

Metastasis is the major cause of cancer treatment failure and it is imperative to develop new antimetastasis therapies. Tumor metastasis is a multistep and complex biological process. Natural compounds derived from medical plants possess diverse structures and broad pharmacological activities and could be used in the treatment of a variety of diseases including cancer.^14^ In this study, we focused on natural compound SG and investigated its inhibitory effect on CRC viability, migration, and metastasis. The results of MMT, colony formation, transwell assay in vitro and xenograft model and metastasis model in vivo indicated that SG could remarkably suppress the viability, migration, and metastasis of LoVo cells.

Tumor cells first detached from the primary lesion, diffused and survived in the circulatory system, and finally colonized and formed metastatic foci in the remote organs.^15^ The viability, motility, and migration ability were essential for tumor cells to detach from the primary lesion. So we investigated the effect of SG on the viability and migration of LoVo cells. The obtained results demonstrated that SG inhibited the viability and migration of LoVo cells in vitro. We also established a metastasis mice model by tail vein injection of LoVo cells and investigated the antimetastatic effect of SG in vivo. The results showed that the SG‐treated group strikingly reduced the number of metastatic nodules and increased the hepatic and pulmonary weight of mice compared with the metastasis model group, indicating a satisfactory antimetastatic effect of SG.

In the early stage, tumor cells are relatively stationary with poor migration ability under the action of extracellular matrix and adhesion molecules such as cadherin. As polarized epithelial cells, these tumor cells could acquire migration ability through the EMT process. EMT always causes high capacity for invasion and migration in tumor, which limits total surgical resection and conduces therapeutic resistance, finally causing tumor recurrence.^16^ The suppression of E‐cadherin results in the loss of cell polarity and adhesion stability and the destruction of epithelial tissue integrity, resulting in the occurrence of EMT. N‐cadherin is a sort of cadherin with weak cellular bond, the expression of which is invariably increased during the EMT process, releasing signals that accelerate cell migration.^16^ Inducing transcription of Snail and secretion of MMPs (MMP2 and MMP9) also occur in EMT progression.^17^, ^18^ Our results of western blotting and immunohistochemistry assay demonstrated that SG remarkably upregulated the expression of E‐cadherin and reduced the protein level of N‐cadherin, Snail, and MMPs (MMP2 and MMP9).

Growing evidence indicates that a majority of CRC patients carry loss‐of‐function or activating mutation in genes that encode the critical members of Wnt/β‐catenin pathway, aberrant activation of which will promote EMT and eventually contributes to tumor development.^19^ In the resting state, β‐catenin is degraded via the destruction complex constituted by APC, AXIN1/2, GSK‐3β, and CK1α. On the contrary, once Wnt ligand binds to its receptor complex, the formation of destruction complex is disturbed and β‐catenin degradation is inhibited due to the phosphorylation of LRP. As a result, active β‐catenin accumulates in the cytoplasm, followed by translocation into the nucleus, where it serves as a transcriptional co‐activator to bind with T‐cell factor/lymphoid enhancer factor and finally induces the expression of target oncogenes including Snail and MMPs.^20^ XAV‐939, one of the Wnt/β‐catenin signaling inhibitor, stabilized AXIN by suppressing the poly‐ADP‐ribosylating enzymes tankyrase 1 and tankyrase 2, thereby promoting β‐catenin degradation.^21^ Here, we found that concurrent treatment with SG and XAV939 augmented the prohibitive effect of SG on LoVo cells migration. SG upregulated the expression of AXIN1 and p‐β‐catenin, and suppressed the level of APC, phosphorylation of GSK‐3β, and translocation of β‐catenin. These indicated the suppression of Wnt/β‐catenin pathway by SG. XAV939 treatment increased the phosphorylation of β‐catenin and the protein level of E‐cadherin, and suppressed the expression of N‐cadherin, MMP2, and MMP9, which confirmed that the suppression of Wnt/β‐catenin signaling could regulate the EMT‐related molecules. Moreover, co‐administration of SG and XAV939 demonstrated a synergistic inhibitory effect on the regulation of β‐catenin and EMT‐related molecules. These results indicate that SG could decrease β‐catenin expression by promoting its phosphorylation and suppress its translocation in the nucleus, leading to the regulation of EMT‐related molecules and inhibition of EMT in LoVo cells.

## 5 CONCLUSIONS

**FIGURE 8:**
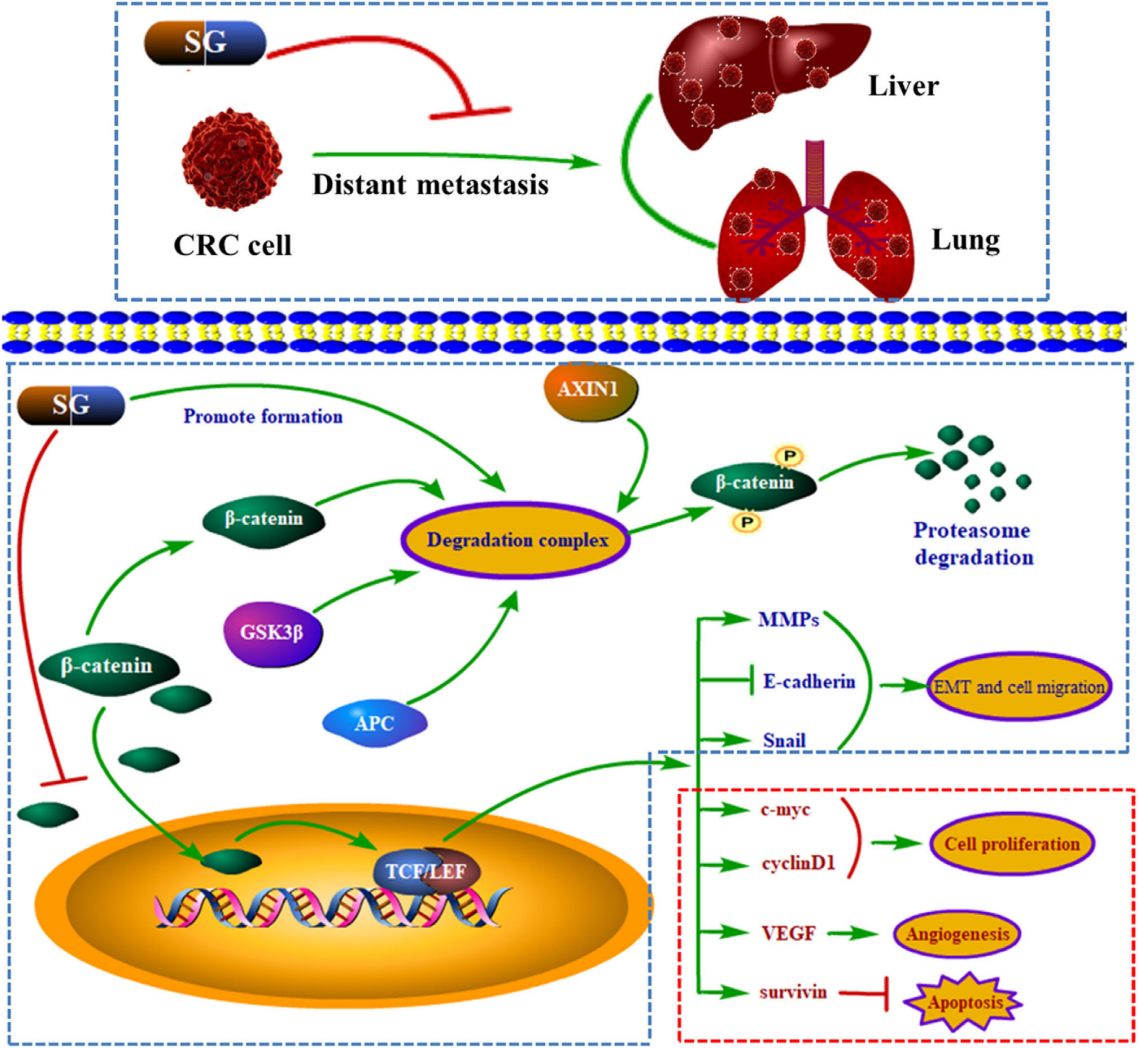
Molecular mechanisms of SG inhibited the metastasis of colorectal carcinoma (CRC) with the potential association of clinical therapies

Here, we established xenograft and metastasis models of CRC cells and investigated the inhibitory effect of SG on CRC viability, migration, and metastasis. And the underlying mechanism was further explored. The data obtained in this research indicated that SG could suppress the CRC LoVo cells migration in vitro and the metastasis to lung and liver in vivo. The potential mechanism attributed to the suppression of the Wnt/β‐catenin signaling by SG, which further upregulated the level of E‐cadherin and decreased the expression of N‐cadherin, Snail, MMP2, and MMP9, and finally prevented EMT (Figure 8). SG holds promising potential in antimetastasis of CRC.

## AUTHORS’ CONTRIBUTIONS

Yanmin Zhang designed the study and interpreted the data, and edited the manuscript; Man Zhu wrote the manuscript, designed the study, and analyzed and interpreted the data; Zhengyan Gong and Qing Wu performed the experiments and interpreted the data; Xianpeng Shi and Qi Su prepared the figures and edited the manuscript. All authors approved the final version of the manuscript.

## CONFLICT OF INTEREST

The authors declare that they have no conflict of interest.

## Data Availability

The data used in the current study are available from the corresponding author on reasonable request.
